# Validity of Child Sleep Diary Questionnaire among Junior High School Children

**DOI:** 10.2188/jea.14.1

**Published:** 2005-03-18

**Authors:** Alexandru Gaina, Michikazu Sekine, Xiaoli Chen, Shimako Hamanishi, Sadanobu Kagamimori

**Affiliations:** 1 Department of Welfare Promotion and Epidemiology, Toyama Medical and

**Keywords:** validity, questionnaire, sleep, schoolchildren, Actiwatch^®^

## Abstract

BACKGROUND: The validity of sleep quality and quantity indices as reported by schoolchildren has not been established. The purpose of this study is to evaluate the relationship between subjective sleep habits estimation and objective measurement data in schoolchildren.

METHODS: The study consisted of 42 healthy junior high school children aged 13-14. Sleep log information was gathered over 7 consecutive days, using a sleep-monitoring device (Actiwatch^®^) and a questionnaire which covered the following aspects for sleep quality and quantity: bed time, sleep latency, sleep start, sleep end, wake up and assumed sleep length. The means of the sleep indices for 5 weekdays were used for analysis. Pearson’s correlation coefficients and paired t-tests were used to evaluate the correlation and difference between subjective and objective sleep parameters.

RESULTS: The correlation coefficient between subjective and objective records was 0.49 (p<0.001) for sleep latency, 0.99 (p<0.001) for sleep start time, 0.99 (p<0.001) for sleep end time, and 0.97 (p<0.001) for assumed sleep length. The difference between subjective and objective records was 7.67 min (95% confidence interval [CI]: 4.64-10.71) for sleep latency, and 0:02 min (95% CI: −0:01-0:05) for sleep start time, 0:02 min (95% CI: 0:01-0:03) for sleep end time, and 8.19 min (95% CI: 4.93-11.45) for assumed sleep length.

CONCLUSIONS: Although children tended to overestimate sleeping hours, the correlation between subjective and objective sleep indices except sleep latency was quite high. Thus, children’s sleep questionnaire can be applied to surveys for sleep habits screening.

The assessment of sleep patterns is widely performed in sleep studies. From the earliest times of epidemiology, questionnaires were used as a basic instrument for data collecting and screening.^1^^,^^2^ Due to the practical and economical aspects, self-reported sleeping habit questionnaires remain to be the most requested and widely used method.^3^,^4^ Subjective sleep assessments, in our study daily sleep diaries, are agreeable for participants, require minimum supervision and can be easily completed. Objective methods, like actigraphy, provide accurate information on the sleep-wake patterns. Actigraphy has been established as a valid method in assessment of the sleep-wake patterns.^5^^,^^6^

However, the validity of sleep questionnaire is not well established. To our knowledge, there are few papers regarding the validation and differences between subjective and objective findings. Sekine et al.^7^ investigated this aspect on preschool children and our present report focused on the validity of the questionnaire for junior high school children during schooldays.

## METHODS

### Subjects

A letter of intention was sent to the parents of 141 children, all boys aged 13-14 years, who are studying in two middle schools in Toyama city. The study was confined only to the boys, because of measurement convenience and recommendation of the school administration. Of these, 47 agreed to participate and gave written informed consent. For the reason that after experiment all subjects received book coupons (money equivalent) as remuneration, mainly children from low-income families consented to be subjects. Parents concerned about children’s free time (experiment took place after lessons) refused to allow children to participate. Besides, parents and children who displayed substantial interest to sleep results decided to participate in our experiment. Data from three children was not successfully collected, due to subjective (forgot to wear Actiwatch^®^) and objective (artifact during reading and recording) reasons. Also, two children complained about adverse allergic reactions (not related with present experiment) and we stopped measurement. The final group consisted of 42 children, with an average age of 14.2 years (standard deviation [SD]: 0.3). At the time of experiment, all children were in good health condition. School start time was 8.30 am from Monday to Friday.

### Anthropometric measurement

On the first day of the experiment, the children wore light clothes and we recorded all anthropometric data: the height of children was measured using a stadiometer, to the nearest 0.1 cm, the weight was measured using a balance scale, to the nearest 0.1 kg. Body mass index (BMI: the weight in kg divided by the squared height in meters) was calculated as an index of obesity. All anthropometric measurements were conducted twice and the mean was used in analysis. The means of height and weight were 166.9 cm (SD: 5.6) and 56.4 kg (SD: 6.3), and the mean of BMI was 20.2 kg/m^2^ (SD: 2.0).

### Evaluation of sleeping habits

Due to the purpose of this experiment, the sleeping habits were evaluated in two ways, first subjectively, using questionnaire and daily records of sleeping schedule and second objectively, using Actiwatch^®^monitoring system, a wrist actigraph which can estimate sleep-wake schedule by measurement of activity level.

We used a questionnaire, concerned detailed sleep schedule, which was completed every day by each child and included records of bedtime, sleep latency time, sleep start time, sleep end time, wake up time, and assumed sleep length. Children were instructed to fill in the answers alone, strictly after wake up. Sleep questionnaire data were expressed in hours and minutes. For sleep latency time, we used fixed a six-response category (5, 10, 20, 30, 40, and over 40 minutes). The means of the values from the 5 schooldays were used in the analyses.

In order to record objective parameters of sleeping habits, we used actigraphy, a method used to estimate sleep-wake rhythms by measurement of gross motor activity. Due to many factors, (long term use, require minimal supervision, large spectrum of sleep parameters), Actiwatch^®^ (Mini Mitter Company Inc., Bend, Oregon, USA) monitoring system, a non-intrusive method is the most available for monitoring actual sleep in children. Till now Actiwatch^®^ has been used in a variety of populations^7^ (including children) in order to discover sleep schedule and sleep disturbances.^8^ The American Academy of Sleep Medicine recognizes it as a useful adjunct in the clinical assessment of sleep disorders.^5^ Also, Actiwatch^®^ has been validated using polysomnography as a reference.^6^

Actiwatch^®^ represents a small, lightweight (17g), limb worn, activity-monitoring device. Internal memory and programming allow Actiwatch^®^ to record and keep data for a long period of time. There is an omni-directional sensor inside, which integrates the degree and speed of motion and produces an electrical current that varies in magnitude according to activity’s intensity. The high level of speed and motion produces increase in voltage. The children were instructed to keep Actiwatch^®^ attached for seven consecutive days, on non-writing wrist. All communication with Actiwatch^®^ was accomplished using an Actiwatch^®^ reader, connected to personal computers. Prior to data collection, we made settings (name, age, sex) and record parameters as follow: epoch length 1.0 min and threshold automatic sensitivity (the algorithm automatically scores an epoch as sleep if the total activity value is equal to or less than the threshold sensitivity value calculated by mean score in active period multiplied by K (constant=0.888), and divided by epoch length). Seven days later we collected the information, by entering the bedtime and wake up time manually for every day, and received automatically calculated results according to the algorithm. Below are definitions and algorithm of the parameters used in the present report. Bedtime is the time at which the subject went to bed with intentions to sleep. Researchers must set this parameter manually, according to the sleep diary. Sleep latency is the period of time between bedtime and the sleep start. Sleep start is the time of sleep onset that was searched for the first 10 min periods in which no more than one epoch is scored as mobile. Sleep end is the time of sleep termination. The software examined the 10 min period directly before the wake up time. The last epoch with no movement is scored as the sleep end time. Wake up is the time at which the subject left the bed. This parameter needs to be inputted manually, according to records from the sleep diary. Assumed sleep is the difference in time between the sleep end and the sleep start.

### Statistical analysis

The subjective and objective sleep schedule parameters were compared using Pearson’s correlation coefficients and paired t-tests. All the statistical analyses were performed with SPSS^®^ (10.0 J). The statistical significance level was set at p<0.05.

## RESULTS

**Figure 1.**
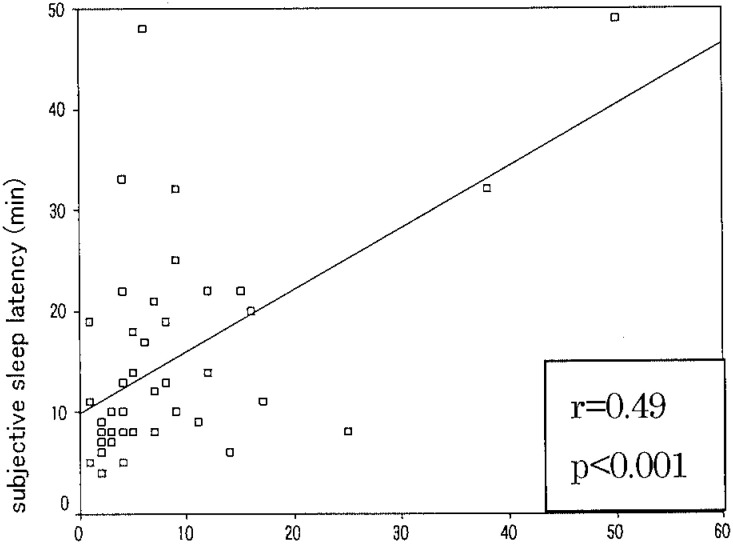
Relationship between subjective and objective measurements of sleep latency time.

**Figure 2.**
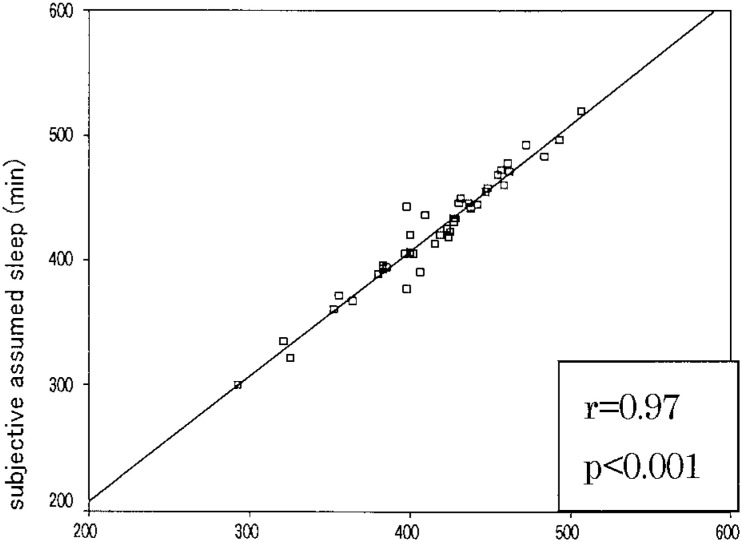
Relationship between subjective and objective measurements of assumed sleep time.

Table 1 shows the mean and SD of the results of correlation coefficients and difference between the subjective and objective sleep parameters. Figures 1 and 2 show the relationships between objective and subjective data of sleep latency and assumed sleep.

**Table 1.** Correlations and differences between subjective and objective sleep parameters

		mean (standard deviation)		Pearson’s correlation coefficient (p value)	difference^†^ (95% confidence interval)

	n	subjective (daily records)	objective (Actiwatch^®^)		
Sleep latency (min)	42	15 (10.7)	7.3 (8.5)	0.49 (<0.001)	7.67 (4.64-10.71)
Sleep start (hr:min)	42	23:49 (0:53)	23:47 (0:52)	0.99 (<0.001)	0:02 (-0:01-0:05)
Sleep end (hr:min)	42	7:00 (0:26)	6:58 (0:26)	0.99 (<0.001)	0:02 (0:01-0:03)
Assumed sleep (min)	42	424.1 (46.4)	415.9 (45.0)	0.97 (<0.001)	8.19 (4.93-11.45)

† : subjective value minus objective value

## DISCUSSION

The principal aim of this report was to assess the validity of subjective sleep habits diary by comparing with objective findings. Subjective records of sleeping hours are highly correlated to objective findings recorded by Actiwatch^®^.

Our findings are in unison with a previous study in young children conducted by Sekine et al.^7^ The relatively low correlation result for sleep latency (r=0.49) with difference 7.67 min has reasonable explanation: (1) Children cannot appreciate exactly the time between going to bed and sleep start. (2) Children try to choose mature pre-sleep behavior; bedtime routine in some subjects includes many activities, like reading, watching television, using mobile phone, and listening to music. All these activities take place in bed and directly influence the time perception of sleep latency. (3) In questionnaire records for sleeping time, we used fixed data for sleep latency. (4) Also, analyzing one-week sleep patterns we observed that often drowsiness occurred before sleep start so children might be confused how to score it. (5) Few subjects have long sleep latency time (long delay in falling asleep) over 20 min, and this factor also affects general results. Even children tend to overestimate sleep latency time, the mean average remain to be 7-8 min, in concordance with other findings.^9^ This is a good indicator for high sleep drive. Based on the present findings, subjective sleep questionnaire records could be used in determining and evaluation of sleep schedule and could be applied in to a large epidemiologic survey.

Present study has several limitations. One of them is that only boys participated in our experiment. Also, we expected a higher rate of participation, but only 1/3 accepted to join our experiment. Children’s behavior related with necessity to use Actiwatch^®^ and to complete sleep dairy questionnaire could introduce some additional sources of observational bias. However, all children were instructed to keep their usual sleep habits schedule. In addition, the self reported sleeping habits were strongly associated with the objective data from Actiwatch^®^. It needs to be mentioned as another limitation of the present study; the high interest of participants in their own sleep habits and excessive attention received from teachers and parents during experiment might exist. All the above factors may affect the accuracy of questionnaire. The validity of subjective sleep habits in a general population might be lower than we found in our study. Although some experiment conditions could compromise the natural sleep settings and observation bias is inevitable, this factor may not be considered to influence the results to a great degree.

In conclusion, subjective sleep habits described subjectively by children are highly correlated with objective findings by Actiwatch^®^. The difference between the subjective vs the objective was small enough to be acceptable in the surveys in sleep habits. Thus subjective evaluation of sleeping habits could be used in a large-scale population survey.
